# Timing, continuity, and energy dose of oral nutritional supplements in older coronary care unit patients: associations with prognostic nutritional index trajectory and hospitalization burden

**DOI:** 10.3389/fnut.2026.1916756

**Published:** 2026-08-13

**Authors:** Shengquan Wang, Yu Zhang, Yanyu Yuan, Chen Huang, Ziwei Chen

**Affiliations:** 1 Department of Clinical Nutrition, Affiliated Hospital of Nantong University, Nantong, Jiangsu, China; 2 Department of Cardiology, Affiliated Hospital of Nantong University, Nantong, Jiangsu, China

**Keywords:** coronary care unit, hospitalization burden, nutritional recovery, oral nutritional supplementation, prognostic nutritional index

## Abstract

**Objectives:**

Older adults account for a disproportionate share of coronary care unit (CCU) admissions, where acute cardiovascular instability, systemic inflammation, and nutritional deterioration converge. This single-center retrospective cohort study explored whether the timing, continuity, and daily energy dose of oral nutritional supplements (ONS) carry distinct associations with nutritional recovery and hospitalization burden in older CCU patients, to generate hypotheses for prospective investigation.

**Methods:**

This single-center retrospective cohort study included 98 CCU patients (median age 80 years, IQR 73–86; 56.1% male) who received nutrition consultation-guided ONS between January 2023 and January 2026. Three delivery characteristics were examined: time from admission to ONS initiation, the ratio of ONS duration to total hospital stay, and average daily energy dose. Prognostic nutritional index (PNI) was tracked at admission, ONS initiation, and discharge or ONS cessation. Multivariable linear regression was used across three hierarchical models, supplemented by restricted cubic spline modeling and inverse probability weighting.

**Results:**

PNI followed a V-shaped trajectory—declining from 37.65 ± 6.07 at admission to 35.24 ± 5.01 at ONS initiation, then rising to 36.26 ± 5.08 at discharge. Earlier initiation was independently associated with shorter hospital stay (*β* = 0.066 per day of delay, 95% CI: 0.043–0.088, *p* < 0.001) and lower total expenses (*β* = 0.056, *p* < 0.001). Greater continuity was similarly associated with reduced burden (*β* = −1.055, *p* < 0.001). Higher daily energy dose was associated with attenuated PNI improvement (*β* = −0.519 per 100 kcal/day, 95% CI: −0.801 to −0.237, *p* < 0.001). Initiation time and continuity were not significantly associated with PNI change.

**Conclusion:**

In this single-center exploratory cohort, the three ONS delivery features did not converge on the same recovery domain. Earlier initiation and sustained continuity were associated with lower hospitalization burden. Higher energy dose was associated with attenuated PNI improvement, a pattern likely reflecting confounding by indication, not a direct adverse effect of ONS energy. These exploratory associations suggest that timing and consistency in ONS delivery may warrant prospective evaluation alongside the decision to supplement. They do not establish causal relationships.

## 1 Introduction

Patients admitted to the coronary care unit (CCU) represent a particularly vulnerable clinical group. Acute cardiovascular instability, systemic inflammation, older age, and reduced oral intake converge to compress physiological reserve. Malnutrition and nutrition-related risk have been consistently linked to adverse cardiovascular outcomes (1–6). This risk is amplified in older adults, who account for a disproportionate share of CCU admissions. Age-related changes (including anorexia of aging, blunted anabolic response to feeding, immunosenescence, and reduced physiological reserve) compound the nutritional vulnerability imposed by critical illness. In this population, nutritional deterioration is not merely a correlate of disease severity but a potentially modifiable driver of recovery trajectory. Yet evidence for how nutritional support should be tailored to older CCU patients remains sparse (7, 8).

Among the tools available to characterize nutritional status, the prognostic nutritional index (PNI) offers particular appeal in older cardiovascular populations. Calculated from serum albumin and peripheral lymphocyte count, PNI serves as a pragmatic composite nutrition-inflammation marker (9). Low PNI has been associated with higher mortality and major adverse cardiovascular events in heart failure and coronary artery disease (9–13). Most prior studies, however, have treated PNI as a static baseline risk marker. This approach is incomplete in older CCU patients, where albumin and lymphocyte counts can shift rapidly under the influence of acute inflammation, fluid redistribution, and nutritional therapy. Longitudinal PNI change during hospitalization may be more informative than a single admission value. It reflects the net balance of catabolic drivers and nutritional repletion, and this balance is particularly tenuous in older adults. Few studies have tracked PNI serially through the CCU stay or examined its relationship with modifiable features of nutritional support delivery.

Oral nutritional supplements (ONS) are a cornerstone of nutritional support in hospitalized patients who can tolerate oral intake. Systematic reviews have demonstrated that ONS can improve nutritional intake, body weight, and functional outcomes in older hospitalized patients (14–18). The evidence base, however, has almost uniformly treated ONS as a simple present/absent variable, overlooking how ONS is actually delivered. When supplementation begins after admission, how continuously it is sustained across the care episode, and what energy level is prescribed may each carry distinct clinical implications. Whether earlier initiation, sustained continuity, or adequate energy dosing is associated with better nutritional recovery could guide ONS prescribing in the CCU. Whether and how these delivery features are associated with nutritional recovery and hospitalization burden in older CCU patients remains unknown.

This study examined the associations of three ONS delivery characteristics—initiation time, continuity (operationalized as the duration-to-stay ratio), and daily energy dose—with PNI trajectory and hospitalization burden in a cohort of older CCU patients who received nutrition consultation-guided ONS.

## 2 Materials and methods

### 2.1 Study design and setting

This single-center retrospective cohort study analyzed data from the CCU of a tertiary hospital. Consecutive patients admitted between January 2023 and January 2026 who received a clinical nutrition consultation and subsequent ONS were included. The requirement for informed consent was waived by the Ethics Committee, given the retrospective design and use of de-identified data. Reporting follows the STROBE statement for observational studies (19).

### 2.2 Participants

**Figure 1:**
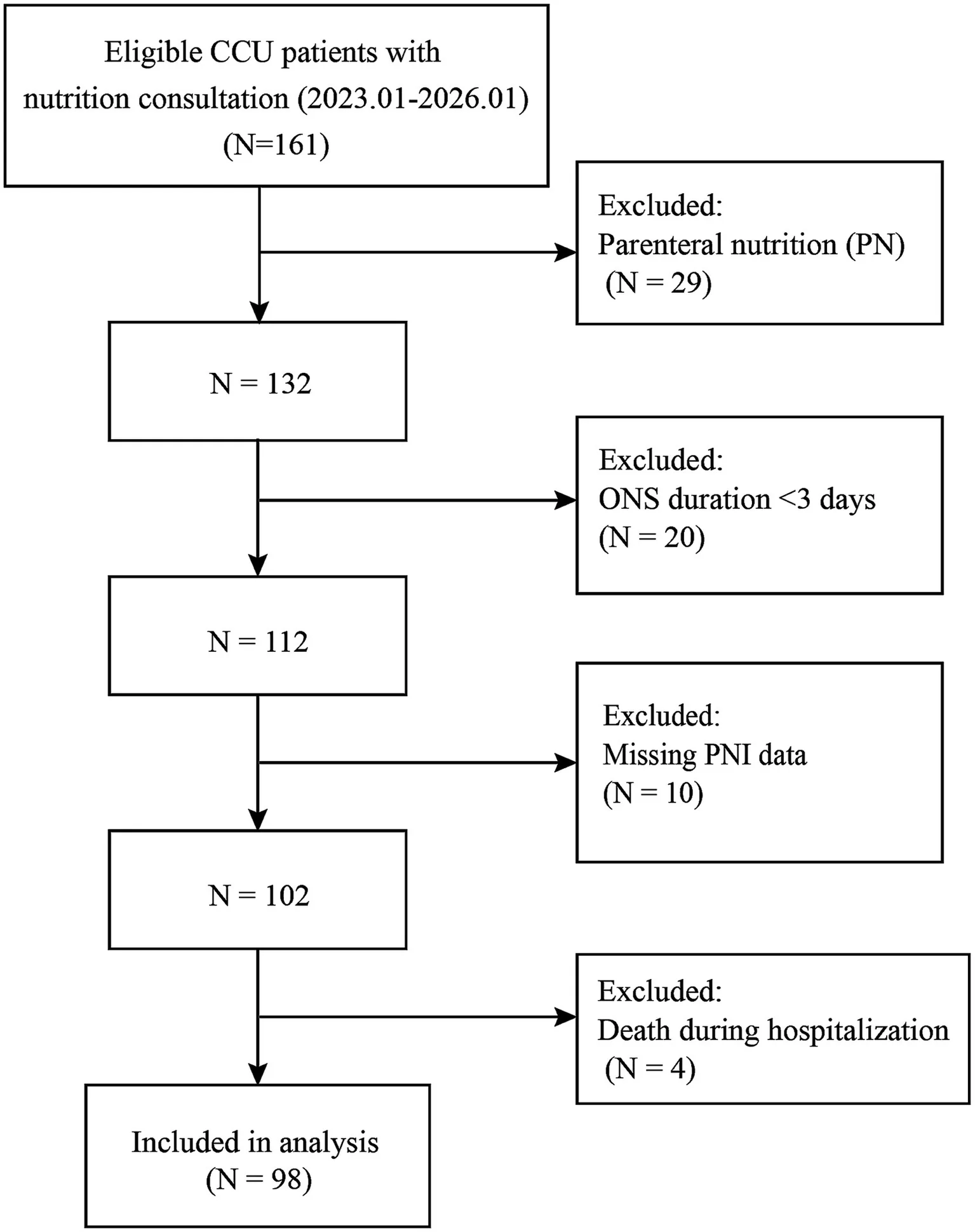
Flow chart of patient selection. CCU, Coronary Care Unit; ONS, Oral Nutritional Supplement; PNI, Prognostic Nutritional Index.

Consecutive patients admitted to the CCU who received a clinical nutrition consultation followed by an ONS prescription were eligible. Exclusion criteria were parenteral nutrition, ONS treatment for fewer than 3 days, insufficient PNI data to calculate the PNI outcomes, and death during hospitalization. The patient selection process is shown in Figure 1. No *a priori* sample size calculation was performed; all eligible patients admitted during the study period were consecutively included

### 2.3 ONS delivery characteristics

Three delivery characteristics were defined to capture distinct dimensions of the ONS process: ONS initiation time (days), the interval between CCU admission and the first day of ONS prescription; ONS duration-to-total-stay ratio, the ratio of ONS use days to total hospital length of stay; and average daily ONS energy dose (kcal/day), total prescribed ONS energy divided by the number of ONS use days.

### 2.4 ONS products

Six commercial powder formulations from three manufacturers were used (Bangshidi [Guangdong] Medical Food Co., Ltd.; Yabao Pharmaceutical Group Co., Ltd.; Xi’an Libang Clinical Nutrition Co., Ltd.). Each was reconstituted to 200 mL per serving for oral or nasogastric administration. Energy density ranged from 0.8 to 1.0 kcal/mL, protein from 8.0 to 10.8 g per serving, and dietary fiber from 0 to 2.7 g per serving. All products were commercially available fixed-formulation products. Formulation choice was guided by individual nutritional assessment.

### 2.5 Outcome measures

The primary nutritional outcome was the change in PNI during the ONS intervention period (ΔPNI_ONS), defined as PNI at discharge or ONS cessation minus PNI at ONS initiation. The secondary nutritional outcome was the net change in PNI across the entire hospitalization (ΔPNI_total). PNI was calculated as serum albumin (g/L) + 5 × lymphocyte count (10^9^/L). Hospitalization burden outcomes included total hospital length of stay (days) and total hospitalization expenses (CNY), both log-transformed to reduce distributional skewness.

### 2.6 Covariates

Covariates included age, sex, admission diagnosis category, cardiac disease severity, diabetes mellitus, mechanical ventilation, continuous renal replacement therapy, PNI at ONS initiation (or admission PNI for ΔPNI_total models), log-transformed N-terminal pro-B-type natriuretic peptide, log-transformed C-reactive protein, and human albumin solution administration. Cardiac disease severity was assessed using the New York Heart Association (NYHA) functional classification or the Killip classification, as appropriate to the clinical diagnosis. Because these systems are not directly equivalent, no class-by-class mapping was attempted. Instead, both systems were collapsed into a binary variable: classes I and II were categorized as mild, and classes III and IV as severe. This harmonized definition allowed disease severity to be analyzed consistently across diagnostic groups. Human albumin solution (HAS) administration was defined as any albumin infusion during the index hospitalization. These covariates were selected a priori based on clinical relevance and prior evidence of their association with nutritional status or outcomes in critically ill cardiovascular populations. Age, sex, and comorbidity burden were included as demographic confounders. Cardiac disease severity and mechanical ventilation captured illness acuity. Log-transformed NT-proBNP was included as a marker of cardiac hemodynamic stress. Log-transformed CRP served as a measure of systemic inflammatory burden. Both may confound the relationship between ONS delivery and nutritional recovery. Human albumin solution administration was included because exogenous albumin directly influences serum albumin, a component of PNI.

### 2.7 Statistical analysis

The analytical strategy was designed to characterize the independent associations of ONS delivery features with outcomes while rigorously addressing confounding. Three hierarchical linear regression models were specified. Model 1 was unadjusted. Model 2 included age, sex, diagnosis category, cardiac severity, diabetes mellitus, mechanical ventilation, and continuous renal replacement therapy. Model 3 further adjusted for PNI at ONS initiation (or admission PNI), log-transformed NT-proBNP, log-transformed CRP, and human albumin solution administration. Daily ONS energy was scaled per 100 kcal/day to produce interpretable coefficients on a clinically meaningful scale.

Model assumptions were assessed with standard regression diagnostics, and restricted cubic spline models with three knots examined potential non-linear associations. Sensitivity was evaluated with HC3 heteroskedasticity-consistent standard errors and inverse probability weighting (IPW), a propensity score-based method that reweights observations to achieve covariate balance between exposure groups. For IPW analyses, ONS exposure variables were dichotomized at the population median; propensity scores were estimated using clinically relevant covariates; average treatment effect weights were applied; and extreme weights were trimmed at the 1st and 99th percentiles. A second propensity model excluding hemoglobin and serum phosphate served as a sensitivity analysis. Hemoglobin and serum phosphate were excluded because both are nutrition-related laboratory parameters. These variables may lie on the causal pathway between ONS delivery and nutritional recovery rather than functioning purely as confounders; their inclusion could therefore introduce overadjustment bias. This alternative model assessed whether the primary IPW estimates were sensitive to the exclusion of potentially endogenous covariates. Pre-specified subgroup analyses tested interaction terms for age, sex, cardiac severity, baseline PNI, C-reactive protein, mechanical ventilation, and diabetes mellitus in the association between ONS initiation time and ΔPNI_ONS. After exclusions, no remaining covariates had missing values; complete-case analysis was therefore applied.

All statistical analyses and figure generation were performed using R version 4.6.0 (R Foundation for Statistical Computing, Vienna, Austria). A two-sided *p* < 0.05 was considered statistically significant. Continuous variables were summarized as mean ± standard deviation or median with interquartile range (IQR), depending on normality of distribution. Categorical variables were summarized as frequency with percentage. Regression estimates are reported with 95% confidence intervals.

## 3 Results

### 3.1 Baseline characteristics

The final cohort included 98 CCU patients who received nutrition consultation-guided ONS. The median age was 80 years (IQR: 73–86 years); 93 of 98 patients (94.9%) were aged ≥60 years, and all patients were older than 50 years. Fifty-five patients (56.1%) were male. Coronary artery disease/acute coronary syndrome and heart failure were the predominant admission diagnoses, and severe cardiac disease was present in most patients. The mean admission PNI was 37.65 ± 6.07. ONS was initiated at a median of 4 days after CCU admission (IQR: 2–7) and continued for a median of 7 days (IQR: 5–10), at a median daily energy dose of 600 kcal/day (IQR: 400–800). Detailed demographic, clinical, nutritional, and ONS-related characteristics are presented in Table 1.

**Table 1:** Baseline characteristics of CCU patients stratified by admission PNI.

Variable	Overall (*N* = 98)	PNI at admission < 37.9 (*N* = 47)	PNI at admission ≥ 37.9 (*N* = 51)	*p*-value
A. Demographics and baseline				
Age, years	80.00 [73.00, 86.00]	80.00 [75.50, 85.50]	80.00 [72.50, 86.50]	0.969
Age group				0.503
≥70 years	83 (84.7%)	41 (87.2%)	42 (82.4%)	–
<70 years	15 (15.3%)	6 (12.8%)	9 (17.6%)	–
Sex				0.800
Male	55 (56.1%)	27 (57.4%)	28 (54.9%)	–
Female	43 (43.9%)	20 (42.6%)	23 (45.1%)	–
Primary diagnosis				0.591
HF	43 (43.9%)	21 (44.7%)	22 (43.1%)	–
CAD/ACS	48 (49.0%)	21 (44.7%)	27 (52.9%)	–
AR	3 (3.1%)	2 (4.3%)	1 (2.0%)	–
Others	4 (4.1%)	3 (6.4%)	1 (2.0%)	–
Cardiac severity				0.312
Severe	66 (67.3%)	34 (72.3%)	32 (62.7%)	–
Mild	32 (32.7%)	13 (27.7%)	19 (37.3%)	–
B. Nutrition at admission				
Albumin, g/L	32.91 ± 5.24	29.47 ± 3.31	36.09 ± 4.67	**<0.001**
Lymphocyte count, ×10^9^/L	0.80 [0.52, 1.23]	0.63 [0.35, 0.87]	1.05 [0.69, 1.58]	**<0.001**
PNI	37.65 ± 6.07	32.83 ± 3.58	42.09 ± 4.22	**<0.001**
C. ONS treatment characteristics				
Initiation time, days	4.00 [2.00, 7.00]	4.00 [2.00, 7.00]	5.00 [2.00, 7.00]	0.559
Duration, days	7.00 [5.00, 10.00]	7.00 [5.00, 10.00]	8.00 [5.00, 10.50]	0.980
Duration-to-stay ratio	0.64 [0.44, 0.86]	0.67 [0.46, 0.86]	0.60 [0.44, 0.80]	0.328
Daily energy dose, kcal/day	600.00 [400.00, 800.00]	600.00 [400.00, 800.00]	600.00 [400.00, 800.00]	0.565
D. Laboratory at ONS initiation				
Hemoglobin, g/L	106.70 ± 24.75	100.66 ± 23.64	112.27 ± 24.67	**0.020**
C-reactive protein, mg/L	21.42 [7.05, 58.00]	31.40 [18.20, 85.25]	10.50 [4.12, 29.80]	**<0.001**
Creatinine, μmol/L	129.50 [98.00, 193.00]	141.00 [105.00, 213.00]	119.00 [93.50, 188.00]	0.293
ALT, U/L	23.00 [13.00, 56.25]	26.00 [12.50, 47.00]	23.00 [14.00, 71.00]	0.524
Phosphate, mmol/L	1.14 [0.91, 1.42]	1.18 [1.04, 1.46]	1.09 [0.88, 1.41]	0.395
NT-proBNP, pg/mL	8475.00 [2935.00, 19,100.00]	8620.00 [3660.00, 21,100.00]	7900.00 [2625.00, 17,950.00]	0.520
E. Comorbidities and interventions				
Mechanical ventilation				0.480
Yes	20 (20.4%)	11 (23.4%)	9 (17.6%)	–
No	78 (79.6%)	36 (76.6%)	42 (82.4%)	–
Diabetes mellitus				0.467
Yes	37 (37.8%)	16 (34.0%)	21 (41.2%)	–
No	61 (62.2%)	31 (66.0%)	30 (58.8%)	–
CRRT				0.889
Yes	13 (13.3%)	6 (12.8%)	7 (13.7%)	–
No	85 (86.7%)	41 (87.2%)	44 (86.3%)	–
Human albumin use				0.310
Yes	15 (15.3%)	9 (19.1%)	6 (11.8%)	–
No	83 (84.7%)	38 (80.9%)	45 (88.2%)	–
F. Hospitalization burden				
Total hospital stay, days	12.00 [9.00, 18.75]	12.00 [7.50, 16.50]	11.00 [10.00, 21.00]	0.845
Total expenses, CNY	32,939.10 [22,980.03, 53,453.79]	34,392.03 [22,636.58, 48,510.76]	30,472.18 [23,456.65, 58,608.90]	0.960
ONS expenses, CNY	343.00 [160.62, 625.50]	388.00 [158.00, 628.00]	326.00 [185.00, 621.00]	0.915
ONS cost ratio, %	0.9% [0.5%, 1.8%]	1.0% [0.6%, 1.7%]	0.9% [0.5%, 2.1%]	0.853

PNI at admission groups was divided by the median value. The cut-off values are displayed in the column headers. Continuous variables: normally distributed data are presented as Mean ± SD; non-normally distributed data are presented as Median [IQR]. Normality was tested by Shapiro–Wilk test (alpha = 0.05) based on the overall population. Categorical variables are presented as *n* (%). For ONS cost ratio, values are presented as percentage (%) after multiplying by 100.

Continuous variables: independent *t*-test for normally distributed data and Mann–Whitney *U* test for non-normally distributed data. Categorical variables: Chi-square test; Fisher exact test was used when expected frequency < 5.

*p* < 0.05 was considered statistically significant. Bold values indicate statistical significance (*p* < 0.05).

PNI, Prognostic Nutritional Index; ONS, Oral Nutritional Supplement; CCU, Coronary Care Unit; CRRT, Continuous Renal Replacement Therapy; HF, Heart Failure; CAD/ACS, Coronary Artery Disease/Acute Coronary Syndrome; AR, Aortic Regurgitation; ALT, Alanine Aminotransferase; NT-proBNP, N-terminal pro-B-type Natriuretic Peptide; CNY, Chinese Yuan.

Patients were stratified according to the median admission PNI value (37.9) to provide descriptive context regarding baseline nutritional-inflammatory status, rather than to define a clinical cutoff. Compared with patients above the median PNI, those below had lower hemoglobin (100.66 ± 23.64 vs. 112.27 ± 24.67 g/L, *p* = 0.020) and markedly higher C-reactive protein (median 31.40 vs. 10.50 mg/L, *p* < 0.001; Table 1). These differences reflect greater anemia and systemic inflammation in the lower-PNI group.

### 3.2 PNI trajectory during hospitalization

**Figure 2:**
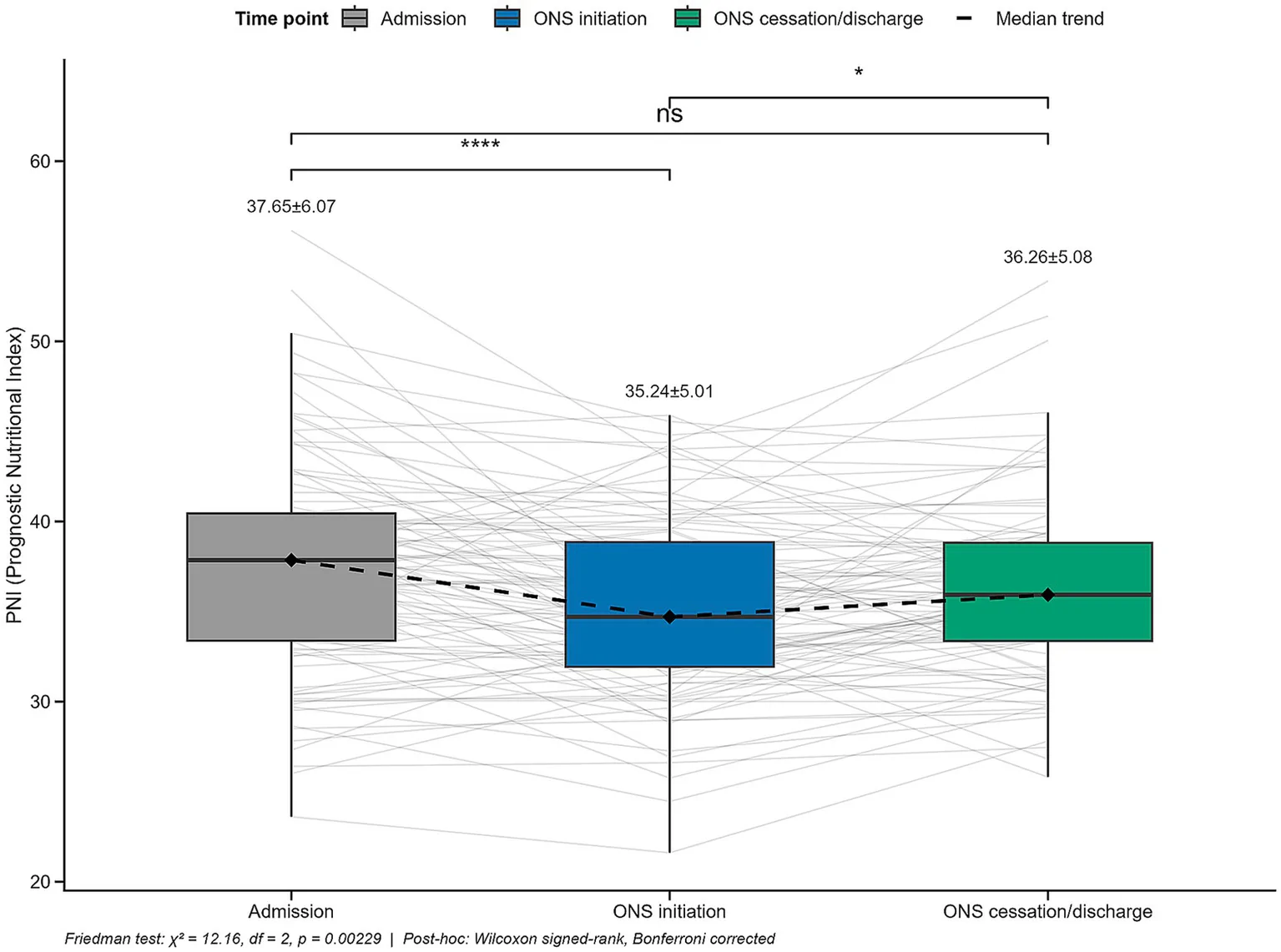
Dynamic trajectory of the Prognostic Nutritional Index across three time points. PNI was measured at admission, ONS initiation, and ONS cessation/discharge in 98 patients. Boxes represent the interquartile range, central horizontal lines indicate medians, and whiskers extend to the most extreme values within 1.5 times the interquartile range. Labels above the boxes report mean ± standard deviation. Gray lines represent paired individual patient trajectories, and the dashed black line with diamond markers connects the median values. Significance brackets indicate Bonferroni-corrected paired Wilcoxon signed-rank comparisons. ΔPNI_ONS = PNI at ONS cessation/discharge − PNI at ONS initiation; ΔPNI_total = PNI at ONS cessation/discharge − PNI at admission. PNI, Prognostic Nutritional Index; ONS, Oral Nutritional Supplement.

Longitudinal PNI changes are shown in Figure 2. Mean PNI fell from 37.65 ± 6.07 at admission to a nadir of 35.24 ± 5.01 at ONS initiation, then rose to 36.26 ± 5.08 by discharge or ONS cessation (ΔPNI_ONS: 1.01 ± 4.39; ΔPNI_total: −1.39 ± 5.89). The Friedman test confirmed significant temporal variation (*χ*^2^ = 12.2, df = 2, *p* = 0.002). Bonferroni-corrected Wilcoxon tests showed a marked decline from admission to ONS initiation (adjusted *p* = 4.65 × 10^−5^), followed by a significant rise during the ONS period (adjusted *p* = 0.030). At discharge, PNI no longer differed significantly from the admission value (adjusted *p* = 0.128), although it had not numerically returned to baseline.

### 3.3 ONS delivery characteristics and PNI changes

Among the three ONS delivery characteristics, only daily energy dose was independently associated with PNI improvement. In the fully adjusted model, each additional 100 kcal/day corresponded to attenuated PNI recovery during the ONS intervention period (*β* = −0.519, 95% CI − 0.801 to −0.237, *p* < 0.001; Table 2). This association persisted in IPW analysis (ATE = −3.36, 95% CI − 5.13 to −1.59, *p* < 0.001; Supplementary Table 2). Neither initiation time nor the duration-to-stay ratio was significantly associated with ΔPNI_ONS or ΔPNI_total after full adjustment (Table 2). Results were consistent across Models 1–3 (Supplementary Table 1).

**Table 2:** Multivariable-adjusted associations of ONS delivery characteristics with nutritional recovery and hospitalization burden.

ONS characteristic	ΔPNI_ONS *β* (95% CI), *p*	ΔPNI_total *β* (95% CI), *p*	log(Hospital stay) *β* (95% CI), *p*	log(Total expenses) *β* (95% CI), *p*
Initiation time of ONS, d	−0.144 (−0.331, 0.044), 0.131	−0.140 (−0.358, 0.077), 0.203	0.066 (0.043, 0.088), <0.001	0.056 (0.024, 0.088), <0.001
Duration ratio of ONS	2.386 (−1.179, 5.951), 0.187	2.859 (−1.263, 6.980), 0.171	−1.055 (−1.505, −0.604), <0.001	−0.978 (−1.584, −0.371), 0.002
ONS energy dose, 100 kcal/d	−0.519 (−0.801, −0.237), <0.001	−0.468 (−0.804, −0.133), 0.007	0.050 (0.009, 0.091), 0.018	0.058 (0.005, 0.111), 0.032

Values are *β* (95% confidence interval), *p* value from multivariable linear regression (Model 3).

Model 3 adjusted for age, sex, primary diagnosis, cardiac severity, diabetes mellitus, mechanical ventilation, CRRT, PNI at ONS initiation (for ΔPNI_ONS) or PNI at admission (for ΔPNI_total), log(NT-proBNP), log(CRP), and HAS.

ΔPNI_ONS = PNI at ONS cessation/discharge − PNI at ONS initiation; ΔPNI_total = PNI at ONS cessation/discharge − PNI at admission.

ONS, Oral Nutritional Supplement; PNI, Prognostic Nutritional Index; CI, Confidence Interval; CRRT, Continuous Renal Replacement Therapy; NT-proBNP, N-terminal pro-B-type Natriuretic Peptide; CRP, C-Reactive Protein; HAS, Human Albumin Solution.

### 3.4 ONS delivery characteristics and hospitalization burden

**Figure 3:**
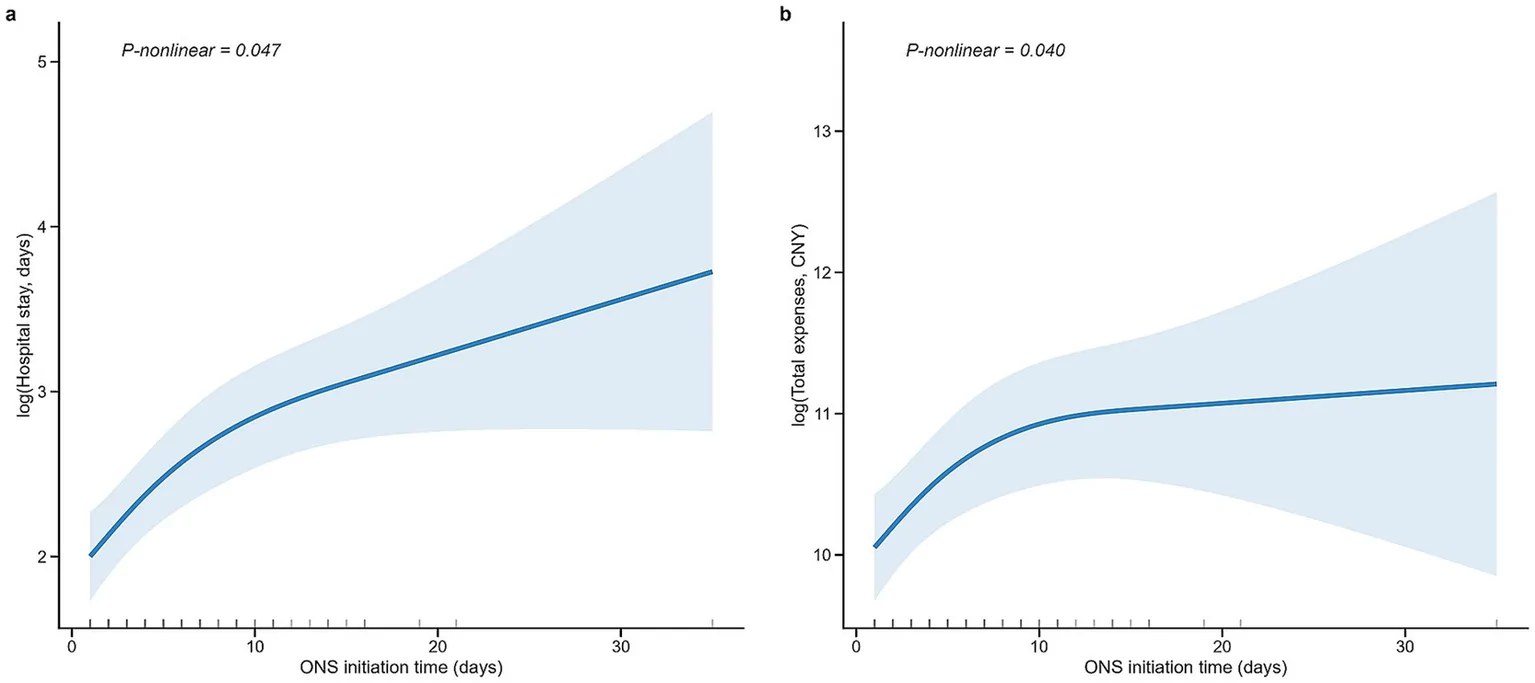
Restricted cubic spline (RCS) dose–response curves for ONS initiation time and hospitalization burden. Panel **(a)**: Log-transformed total hospital stay. Panel **(b)**: Log-transformed total expenses. Solid blue lines indicate predicted values; shaded bands represent 95% confidence intervals. Models adjusted for age, sex, diagnosis, cardiac severity, DM, MV, CRRT, PNI at ONS initiation, log(NT-proBNP), log(CRP), and HAS. RCS, Restricted Cubic Spline; ONS, Oral Nutritional Supplement; PNI, Prognostic Nutritional Index; DM, Diabetes Mellitus; MV, Mechanical Ventilation; CRRT, Continuous Renal Replacement Therapy; NT-proBNP, N-terminal pro-B-type Natriuretic Peptide; CRP, C-Reactive Protein; HAS, Human Albumin Solution.

Earlier ONS initiation was independently associated with shorter hospital stay (*β* = 0.066 per day delay, 95% CI 0.043–0.088, *p* < 0.001) and lower total expenses (*β* = 0.056, 95% CI 0.024–0.088, *p* < 0.001; Table 2). Restricted cubic spline analysis confirmed significant non-linearity, with associations steepening at earlier initiation times (*p*-nonlinear = 0.047 for hospital stay, Figure 3a; *p*-nonlinear = 0.040 for total expenses, Figure 3b).

Greater continuity, as reflected by the duration-to-stay ratio, was also independently associated with shorter stay (*β* = −1.055, 95% CI − 1.505 to −0.604, *p* < 0.001) and lower expenses (*β* = −0.978, 95% CI − 1.584 to −0.371, *p* = 0.002; Table 2). IPW analysis confirmed the associations of earlier initiation and greater continuity with reduced hospitalization burden (Supplementary Table 2).

Daily energy dose showed a modest positive association with longer stay and higher expenses in the fully adjusted model (Table 2), but these findings were not confirmed by IPW analysis (Supplementary Table 2).

### 3.5 Subgroup analyses

Pre-specified subgroup analyses tested whether the association between ONS initiation time and ΔPNI_ONS differed across clinically relevant subgroups. The association varied by diabetes mellitus status (*p* for interaction = 0.014) and sex (p for interaction = 0.036). Earlier ONS initiation was associated with greater PNI improvement in patients with diabetes (*β* = −0.389, *p* < 0.001), but not in those without diabetes (*β* = 0.164, *p* = 0.372). The estimate was negative but nonsignificant in men (*β* = −0.20, 95% CI −0.44 to 0.04, p = 0.104). In women, it was positive but nonsignificant (*β* = 0.23, 95% CI −0.20 to 0.67, *p* = 0.283). No evidence of interaction was found in the remaining subgroups (Supplementary Figure S1).

## 4 Discussion

In this retrospective cohort of older CCU patients, the three ONS delivery characteristics were associated with different clinical domains: earlier initiation and greater continuity with lower hospitalization burden, and higher energy dose with attenuated PNI improvement. The cohort was restricted to patients who received ONS. These findings reflect associations between different ONS delivery strategies, not evidence of ONS effectiveness relative to no supplementation.

The V-shaped PNI trajectory provides important clinical context. Mean PNI fell from 37.65 at admission to a nadir of 35.24 at ONS initiation, before partial recovery to 36.26 at discharge. The post-initiation increase is best understood as recovery from the lowest point, not as net nutritional gain across the entire hospitalization. Prior studies have established that PNI carries prognostic information in cardiovascular disease, including heart failure, coronary artery disease, and acute myocardial infarction (9–13, 20). Recent evidence further supports the value of dynamic PNI assessment (13, 21). At the same time, PNI must be read as a composite nutrition-inflammation marker. Albumin and lymphocyte count are shaped by inflammation, vascular permeability, fluid shifts, infection, and catabolic stress; albumin alone is an unreliable stand-alone nutritional marker during acute illness (22). The V-shaped pattern observed here likely reflects a combination of early illness-related deterioration, subsequent nutritional support, clinical stabilization, and ongoing physiological stress. The trajectory itself—rather than any single measurement—may better capture the nutritional and inflammatory response to ONS delivery.

The independent association between earlier ONS initiation and lower hospitalization burden is clinically instructive. This finding aligns with the AHA scientific statement on malnutrition in acute cardiac conditions, which emphasizes timely nutritional intervention (23), and with ICU nutrition guidance supporting early nutritional support when clinically feasible (16, 24, 25). Direct comparison is constrained because most trials informing current guidelines compared nutritional support against usual care rather than varying timing within a supplemented population. The EFFORT trial demonstrated that individualized nutritional support initiated within 48 h of admission reduced adverse outcomes in medical inpatients and provides the clearest trial-level evidence for early intervention (17). That trial, however, tested a protocol-based binary intervention rather than initiation timing as a continuous variable. Our analysis extends this evidence by showing that, among patients already deemed to require ONS, each additional day of delay was incrementally associated with greater hospitalization burden. Existing guideline-era trials were not designed to detect this dose–response pattern. The non-linear association documented by RCS analysis adds further detail: the steepest gradients occur at the earliest initiation times, suggesting that the first several days after CCU admission may represent a particularly sensitive window. Early ONS may shorten the cumulative period of inadequate intake and support readiness for recovery. An alternative interpretation must also be considered. Earlier initiation may function as a marker of earlier multidisciplinary engagement, better care coordination, or greater clinical stability permitting oral intake. The retrospective design precludes causal attribution.

Greater ONS continuity, operationalized through the duration-to-total-stay ratio, was independently associated with reduced hospitalization burden. This finding is clinically coherent: ONS can only exert its intended effects when delivered consistently. Systematic reviews have reported that ONS can improve clinically relevant outcomes and may be cost-saving in hospitalized populations (18, 26–29). Real-world exposure, however, is shaped by compliance, tolerance, interruptions, and discrepancies between prescribed and actual intake (30, 31)—a gap that the duration ratio may partially capture as a care-process dimension. This construct extends the concept of ONS compliance, which a systematic review has established as a determinant of clinical benefit: actual intake often falls well below prescribed amounts, and higher compliance is associated with greater nutritional and functional gains (30). Whereas compliance reflects patient-level adherence, ONS continuity, operationalized as the duration-to-stay ratio, captures a care-process dimension: the proportion of the hospitalization during which ONS was prescribed and sustained. The existing nutritional support evidence comes largely from trials that treated supplementation as a binary exposure (18, 26). These provide limited data on continuity as a graded predictor, making formal comparison difficult. Caution is warranted. Because the ratio contains total hospital stay in its denominator, mathematical coupling with the outcome cannot be excluded. Before interpreting the energy dose findings, the biological behavior of PNI in this population warrants attention. Future studies should test this finding using fixed-window exposure definitions, interruption frequency, and actual intake records.

The inverse association between energy dose and PNI improvement is the most counterintuitive observation in this analysis and must be interpreted with caution. Confounding by indication is the most plausible explanation. It would be incorrect to conclude that higher ONS energy directly impairs nutritional recovery. A more plausible explanation is confounding by indication: patients prescribed higher ONS energy likely had greater nutritional risk, more severe catabolism, heavier inflammatory burden, or slower recovery trajectories. In addition, an established principle in geriatric nutrition—anabolic resistance—may contribute to this finding. Older adults exhibit a blunted protein synthetic response to feeding compared with younger individuals (32, 33), meaning that higher caloric delivery does not automatically translate into proportionally greater lean tissue accretion or functional recovery (34, 35). Acute critical illness further alters substrate utilization; higher caloric delivery does not guarantee better short-term biological recovery in this population. Contemporary ICU nutrition guidance cautions against uniformly matching energy expenditure in the acute phase, and recent randomized trials have challenged the assumption that more aggressive calorie or protein delivery improves outcomes in critically ill patients (24, 25, 36, 37). Specifically, NUTRIREA-3 compared low versus standard calorie and protein delivery in ventilated adults with shock and found no benefit of higher intake (36); EFFORT Protein similarly reported no advantage of higher protein dosing in critically ill patients at high nutritional risk (37). These trials tested dose variation using enteral or parenteral routes in the ICU. Our observation that higher prescribed ONS energy was associated with less PNI improvement in an older CCU population parallels this finding in a different delivery route and patient profile. Greater caloric delivery did not translate into greater short-term biological recovery in either setting. Direct ONS dose–response comparisons remain scarce: randomized ONS trials in older patients have generally tested fixed-dose regimens against usual care (14, 15, 18), and existing meta-analyses have not identified a clear dose–response gradient for clinical endpoints (18). Our findings suggest that caloric escalation alone is an inadequate short-term recovery strategy when PNI is the outcome in this population—not that ONS energy is harmful.

The divergence between PNI-related and burden-related findings reinforces the delivery-process argument. Earlier initiation and greater continuity were associated with lower hospitalization burden but not with significant PNI improvement. Higher energy dose was associated with attenuated PNI improvement, while its association with burden was not confirmed by IPW analysis. No single delivery feature was uniformly associated with both outcome domains. This pattern points to the complexity of nutritional recovery in older cardiac critical care patients, in whom acute disease physiology, age-related metabolic changes, and nutritional support interact in ways that a single delivery dimension cannot capture. Length of stay and costs may be shaped by workflow, discharge readiness, and care coordination; PNI is more sensitive to inflammatory status, albumin kinetics, and acute disease physiology—all of which may be modulated by age. Clinical heterogeneity across admission diagnoses merits additional consideration. Heart failure, acute coronary syndrome, valvular disease, and other included conditions each carry distinct inflammatory profiles, nutritional requirements, and expected hospitalization courses. Although diagnosis category was adjusted for in all models, residual within-category variation in disease severity may contribute to the divergence between PNI and burden findings. Future research should combine dynamic nutrition-inflammation markers with geriatric assessment—including frailty and sarcopenia measures (38–41), actual dietary intake, inflammatory trajectories, functional measures, and post-discharge outcomes including mortality and readmission. The delivery-process framework advanced here requires prospective validation with multi-domain endpoints in older hospitalized populations.

Several limitations merit consideration and constrain the conclusions that can be drawn from this study. This was a single-center retrospective cohort of 98 patients, and residual confounding cannot be excluded despite multivariable adjustment, robust standard errors, RCS analysis, and IPW. The findings should therefore be interpreted as exploratory and hypothesis-generating rather than definitive. Prospective multicenter cohorts are needed before these associations can inform clinical practice. Factors such as appetite, swallowing function, frailty, and clinician decision-making may have influenced both ONS delivery and outcomes. Frailty and sarcopenia—central constructs in geriatric nutrition research (38–40)—were not formally assessed, and their absence limits inference about whether ONS delivery effects are modified by baseline physiological vulnerability. Because all included patients received ONS, the study cannot determine whether ONS itself is superior to no ONS. PNI is an inflammation-sensitive composite marker; changes in its components may partially reflect illness dynamics rather than nutritional repletion. The analyses relied on prescribed ONS energy, not measured actual intake, which introduces potential exposure misclassification. Adherence to oral nutritional supplements is often suboptimal, particularly in older hospitalized patients, in whom taste fatigue, gastrointestinal intolerance, and polypharmacy interactions are well-documented barriers to compliance (29, 30, 40). The recorded energy dose may overestimate true nutritional exposure in some patients. ONS formulations were not standardized across patients. The six products varied in protein, energy density, fat, and fiber content, and these differences may have influenced nutritional recovery independently of prescribed energy dose. Product-specific effects could not be isolated because formulation choice was guided by clinical indication. The duration-to-total-stay ratio is clinically intuitive but methodologically vulnerable. Because total hospital stay enters both the ratio denominator and the primary outcome, mathematical coupling may partly inflate the observed association between continuity and hospitalization burden. The present design cannot separate this mechanical contribution from a true effect of ONS continuity. The delivery-process framework described here requires prospective validation with actual intake measurement, fixed-window exposure definitions, multi-domain outcomes, and larger, multi-center samples that include geriatric assessment. Because PNI was derived from routine clinical laboratory values, the temporal relationship between blood sampling and albumin infusion could not be determined retrospectively; in patients who received HAS, serum albumin values may reflect a combination of endogenous production and exogenous infusion.

The clinical value of ONS in older CCU patients depends on how it is delivered, not simply on whether it is given. No single delivery feature predicted both recovery domains. When supplementation begins, how continuously it is provided, and what energy level is targeted each carry distinct implications for older adults: timely initiation and sustained delivery were more informative for hospitalization burden, while the inverse energy-dose–PNI association is most plausibly explained by confounding by indication, not a direct detrimental effect of higher caloric intake. These patterns require prospective validation in studies that systematically characterize ONS delivery and incorporate geriatric assessment (38, 39).

## Data Availability

The data are not publicly available due to privacy and ethical restrictions. Requests to access the data should be directed to the corresponding author and will be considered in accordance with applicable ethical and institutional requirements.

## Supplementary material

SUPPLEMENTARY FIGURE S1 Subgroup analysis of the association between ONS initiation time and Delta PNI_ONS. Forest plot showing beta coefficients and 95 percent confidence intervals from multivariable linear regression (Model 3) stratified by age, sex, cardiac severity, baseline PNI, CRP, mechanical ventilation, and diabetes mellitus. *p*-interaction values test for effect modification across subgroups. All subgroup analyses are exploratory and should be interpreted with caution given the non-significant overall association. PNI, Prognostic Nutritional Index; ONS, Oral Nutritional Supplement; CI, Confidence Interval; CRP, C-Reactive Protein.
