# Supplementary material for: Timing, continuity, and energy dose of oral nutritional supplements in older coronary care unit patients: associations with prognostic nutritional index trajectory and hospitalization burden
Source: Front Nutr. 2026 Aug 13;13:1916756. doi: 10.3389/fnut.2026.1916756 (PMC13518111; doi:10.3389/fnut.2026.1916756)
Supplement: Supplementary file 1 [file Table_1.docx]

Supplementary Table 1. Unadjusted and partially adjusted associations of ONS delivery characteristics with all outcomes.

| **ONS Characteristic** | **Model** | **ΔPNI_ONS β (95% CI)** | ***p*** | **ΔPNI_total β (95% CI)** | ***p*** | **log(Hospital stay) β (95% CI)** | ***p*** | **log(Total expenses) β (95% CI)** | ***p*** |
| --- | --- | --- | --- | --- | --- | --- | --- | --- | --- |
| Initiation time of ONS, d | Model 1 (Unadjusted) | -0.029 (-0.201, 0.144) | 0.741 | -0.150 (-0.379, 0.080) | 0.199 | 0.065 (0.047, 0.083) | <0.001 | 0.063 (0.036, 0.089) | <0.001 |
|  | Model 2 (Partially adjusted) | -0.072 (-0.257, 0.113) | 0.439 | -0.141 (-0.395, 0.113) | 0.274 | 0.066 (0.046, 0.085) | <0.001 | 0.049 (0.021, 0.078) | <0.001 |
| Duration ratio of ONS | Model 1 (Unadjusted) | 0.782 (-2.805, 4.370) | 0.666 | 4.094 (-0.652, 8.840) | 0.090 | -1.134 (-1.534, -0.734) | <0.001 | -1.024 (-1.603, -0.444) | <0.001 |
|  | Model 2 (Partially adjusted) | 1.483 (-2.220, 5.186) | 0.428 | 3.737 (-1.327, 8.800) | 0.146 | -1.126 (-1.544, -0.708) | <0.001 | -0.937 (-1.510, -0.364) | 0.002 |
| ONS energy dose, 100 kcal/d | Model 1 (Unadjusted) | -0.523 (-0.810, -0.237) | <0.001 | -0.542 (-0.936, -0.147) | 0.008 | 0.054 (0.017, 0.092) | 0.005 | 0.078 (0.028, 0.128) | 0.002 |
|  | Model 2 (Partially adjusted) | -0.585 (-0.890, -0.281) | <0.001 | -0.620 (-1.054, -0.186) | 0.006 | 0.049 (0.007, 0.090) | 0.022 | 0.050 (-0.002, 0.103) | 0.060 |

Values are β (95% CI), p-value from linear regression. Model 1: unadjusted. Model 2: adjusted for age, sex, diagnosis, cardiac severity, DM, MV, and CRRT. ΔPNI_ONS = PNI at ONS cessation/discharge − PNI at ONS initiation; ΔPNI_total = PNI at ONS cessation/discharge − PNI at admission. Hospital stay and total expenses were log-transformed. See Table 2 for fully adjusted Model 3 results.

Abbreviations: ONS, Oral Nutritional Supplement; PNI, Prognostic Nutritional Index; CRRT, Continuous Renal Replacement Therapy; DM, Diabetes Mellitus; MV, Mechanical Ventilation; CI, Confidence Interval.
