# Supplementary material for: Timing, continuity, and energy dose of oral nutritional supplements in older coronary care unit patients: associations with prognostic nutritional index trajectory and hospitalization burden
Source: Front Nutr. 2026 Aug 13;13:1916756. doi: 10.3389/fnut.2026.1916756 (PMC13518111; doi:10.3389/fnut.2026.1916756)
Supplement: Supplementary file 2 [file Table_2.docx]

Supplementary Table 2. Inverse probability weighting analysis of ONS delivery characteristics on all outcomes.

| **ONS Strategy** | **ΔPNI_ONS ATE (95% CI)** | ***p*** | **ΔPNI_total ATE (95% CI)** | ***p*** | **log(Hospital stay) ATE (95% CI)** | ***p*** | **log(Total expenses) ATE (95% CI)** | ***p*** |
| --- | --- | --- | --- | --- | --- | --- | --- | --- |
| Early ONS initiation (≤ 3 days) | 0.55 (-1.36, 2.47) | 0.573 | 1.42 (-1.10, 3.94) | 0.272 | -0.47 (-0.66, -0.27) | <0.001 | -0.39 (-0.68, -0.10) | 0.009 |
| High ONS duration-to-stay ratio (≥ 0.64) | 1.19 (-0.70, 3.07) | 0.219 | 1.51 (-0.95, 3.96) | 0.233 | -0.45 (-0.64, -0.25) | <0.001 | -0.40 (-0.68, -0.12) | 0.006 |
| High daily ONS energy dose (≥ 600 kcal/day) | -3.36 (-5.13, -1.59) | <0.001 | -3.13 (-5.73, -0.53) | 0.021 | 0.06 (-0.19, 0.31) | 0.643 | 0.16 (-0.29, 0.61) | 0.479 |

ATE, average treatment effect from IPW linear regression. Weights truncated at 1st/99th percentiles.

Propensity scores: the covariates specified in the Methods section. Cut-offs: ≤3 d, study population medians for ratio/energy.

Abbreviations: IPW, Inverse Probability Weighting; ATE, Average Treatment Effect; ONS, Oral Nutritional Supplement; PNI, Prognostic Nutritional Index; CI, Confidence Interval.
